# Nicotinamide-based diamides derivatives as potential cytotoxic agents: synthesis and biological evaluation

**DOI:** 10.1186/s13065-017-0338-5

**Published:** 2017-10-24

**Authors:** Min Peng, Liqiao Shi, Shaoyong Ke

**Affiliations:** 1 Department of Oncology, Renmin Hospital of Wuhan University, Wuhan University, Wuhan, 430060 Hubei China; 2 National Biopesticide Engineering Technology Research Center, Hubei Academy of Agricultural Sciences, Wuhan, 430064 China

**Keywords:** Nicotinamide, Diamides, Synthesis, Lung cancer, Cytotoxic activity

## Abstract

A series of diamides derivatives containing nicotinamide unit were designed, synthesized and evaluated for their potential cytotoxic activities against human cancer cell lines. All the synthesized compounds were characterized using spectroscopic methods mainly including ^1^H NMR, ^13^C NMR and MS. The bio-evaluation results indicated that some of the obtained compounds (such as **4d**, **4h**, **4i**) exhibited good to moderate cytotoxic effects on lung cancer cell lines (NCI-H460, A549, and NCI-H1975), especially, compound **4d** exhibited the highly potential inhibitory activities against NCI-H460 cell line with the IC_50_ values of 4.07 ± 1.30 μg/mL, which might be developed as novel lead compounds for potential cytotoxic agents.

## Background

Lung cancer is the leading disease-related cause of deaths of human population, and which has also seriously threaten the health and life of humans for a long period [1–4]. Although substantial advances have been made in the systemic therapy of solid tumors over the past two decades, most patients obtain only modest benefit from treatment, whereas toxicity is common and the drug resistances rising from the tumor cell mutation challenges current medical therapies [5, 6]. So it is still an unmet medical need to development new drugs having broad therapeutic index (risk/efficacy).

**Fig. 1:**
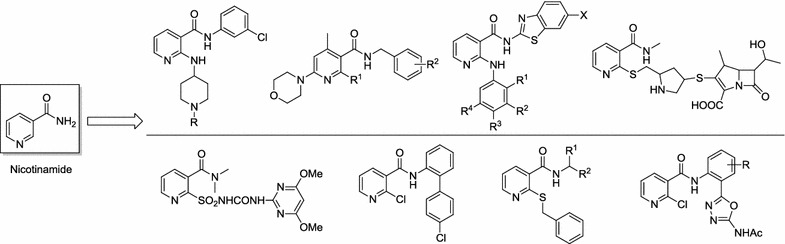
Structures of nicotinamide and its derivatives

Nicotinamide (Fig. 1) is a widely used vitamin [7], and some researches also demonstrated that it might reduce the risk of nonmelanoma skin cancers, and also effective to treat bullous pemphigoid [8]. As a special class of heterocyclic compounds, nicotinamide can be used as accessory reagents for chemotherapy and radiation therapy, and so which received significant attentions for their interesting biological activities. Recently, many nicotinamide derivatives have been reported for their broad pharmacological properties such as anticancer [9–11], anti-angiogenic [9], and antinociceptive effect [12] etc. Meanwhile, some nicotinamide derivatives have also been developed as agrochemicals for their insecticidal, herbicidal, and fungicidal activities [13–18]. In addition, many diamides derivatives have been investigated for their wide range of pharmacological activities including antitumor [19, 20], anti-inflammatory activities, Factor Xa inhibitors [21], CCK1 receptor antagonists [22], and insecticidal and fungicidal activities [23–25] etc., and which all demonstrated this diamide scaffold might be an important pharmacophore for drug discovery. The diverse bioactivity of this class of compounds urges us to construct a series of novel structural variants of diamides derivatives.

**Fig. 2:**
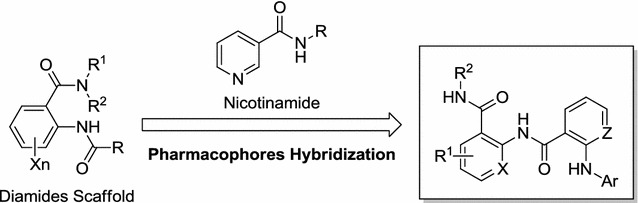
Design strategy of nicotinamide-based diamides derivatives

Thus, based on the aforementioned description, this work focused on the design, convenient synthesis, and cytotoxic evaluation of a series of novel nicotinamide-based diamides derivatives based on pharmacophores hybridization. The nicotinamide and diamide scaffolds have been integrated in one molecule as shown in Fig. 2, and the potential anticancer effects of these prepared compounds were screened against three lung cancer cell lines (NCI-H460, A549, NCI-H1975) and two normal cells (HL-7702, MDCK). The results indicated some of the target molecules might be not a target-specific agent, may behave as a new lead compounds for highly potential cytotoxic agents.

## Results and discussion

### Chemistry

**Scheme 1:**
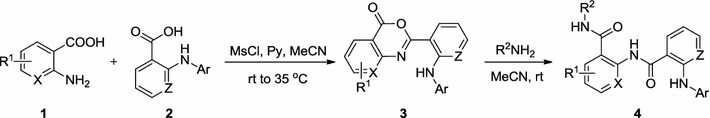
Synthesis of nicotinamide-based diamides derivatives

The general procedures for the preparation of these novel nicotinamide-based diamides derivatives **4a–k** and diamides **4l–o** are outlined in Scheme 1.

**Fig. 3:**
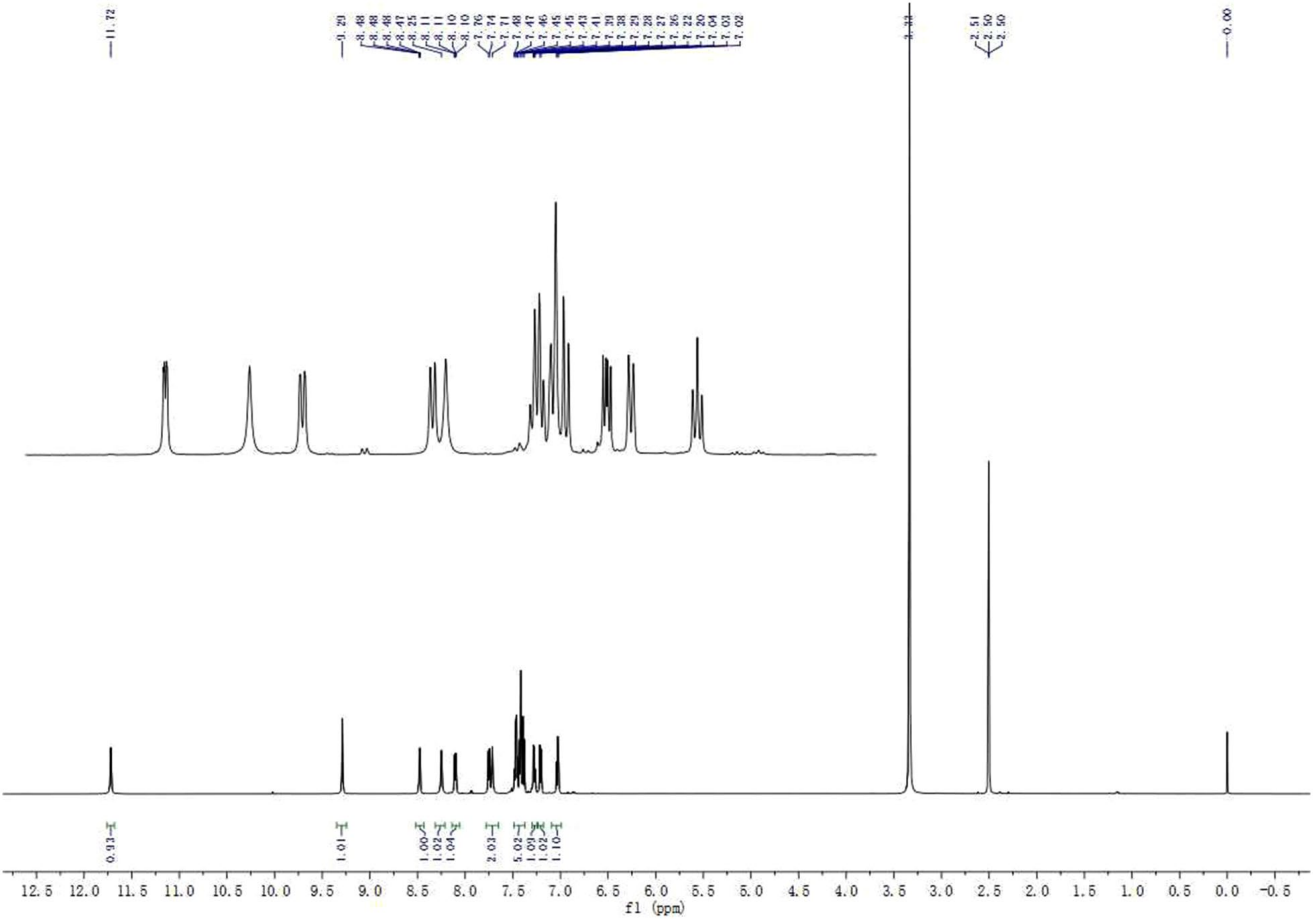
The representative ^1^H NMR spectra analyses for compound **4a**

The key building blocks ortho-amino aryl acid **1** and **2** were selected as starting materials, and which could be conveniently transferred to the corresponding oxazinone heterocyclic intermediates **3** via a classical heterocyclization reaction [13, 20, 23–25] with simple procedures. Then these oxazinones were treated with various amines resulting in the target nicotinamide-based diamides derivatives **4a–k** via nucleophilic substitution reaction. Furthermore, for a few comparative activity measurements of compounds with no nicotinamide moiety, some similar diamide derivatives (where X = Z = C) as shown in Scheme 1 have also been synthesized using the aforementioned similar method. All title compounds gave satisfactory chemical analyses, and the chemical structures of the synthesized compounds were summarized in the part of experimental, and the typical ^1^H NMR spectra analyses for compound **4a** have been shown in Fig. 3.

### Bioassay

**Fig. 4:**
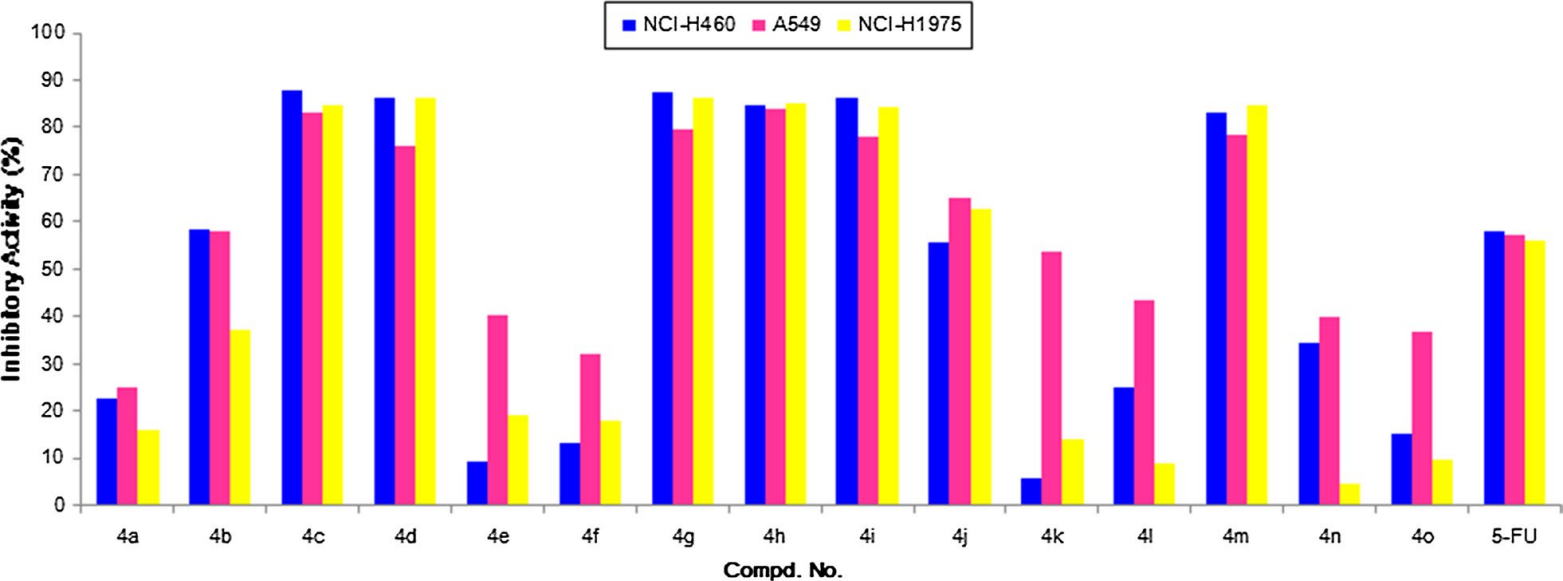
Cytotoxic activities of compounds **4a–k** and **4l–o** at 40 µg/mL. *NCI-H460* human large cell lung cancer cell line, *A549* human lung cancer cell line, *NCI-H1975* human lung cancer cell line, *5-FU* 5-fluorouracil, used as a positive control

All newly prepared nicotinamide-based diamides derivatives **4a–k** and several similar diamides **4l–o** were evaluated for their in vitro cytotoxic effects against NCI-H460 (Human large cell lung cancer cell line), A549 (Human lung cancer cell line), NCI-H1975 (Human lung cancer cell line), HL-7702 (Human normal liver cells), and MDCK (Madin-Darby canine kidney cells) cell lines by the modified MTT (3-(4,5-dimethylthiazol-2-yl)-2,5-diphenyl tetrazolium bromide) assay [26, 27] using 5-FU (5-Fluorouracil) as a positive control. The preliminary results were summarized in Fig. 4.

From the Fig. 4, we can find that some of the nicotinamide-based diamides derivatives indicated moderate to good inhibition activities against these three human lung cancer cell lines. Notably, the compounds **4c**, **4d**, **4g**, **4h**, and **4i** exhibited significant inhibitory activities against all tested cell lines at the concentration of 40 µg/mL. Also, it is interesting to note that compounds **4e**, **4k** and **4l** presented selective cytotoxicity for A549 cell line, and the similar diamide **4m** exhibited good inhibition activities for all tested cell lines.

According to the preliminary bioassay results, some of the title molecules have been demonstrated to exhibit good inhibitory activities against all three lung cancer cell lines, so in order to further explore the potential activities, the IC_50_ values were investigated based on the above cell-based method. The in vitro activities expressed as IC_50_ values for these compounds are described in Table 1.

**Table 1:** In vitro cytotoxic activities of the compounds against human cancer cell lines and normal cells

Entry	Compd no.	Substituents					In vitro cytotoxicity IC_50_^a^ (μg/mL)				
		R^1^	R^2^	X	Z	Ar	NCI-H460	A549	NCI-H1975	HL-7702	MDCK
1	**4a**	H	H	N	CH	3-CF_3_Ph	> 40	> 40	> 40	> 40	> 40
2	**4b**	H	CH_3_	N	CH	3-CF_3_Ph	36.68 ± 2.80	> 40	> 40	> 40	> 40
3	**4c**	H	CH_3_CH(OH)CH_2_	N	CH	3-CF_3_Ph	20.63 ± 1.37	26.31 ± 3.22	27.25 ± 2.76	19.73 ± 0.45	16.98 ± 0.84
4	**4d**	H	CH_3_COCH_2_	N	CH	3-CF_3_Ph	4.07 ± 1.30	13.09 ± 2.45	12.82 ± 1.59	26.87 ± 0.95	13.45 ± 0.29
5	**4e**	3-CH_3_-5-Cl	H	C	N	3-CF_3_Ph	> 40	> 40	> 40	> 40	> 40
6	**4f**	3-CH_3_-5-Cl	CH_3_	C	N	3-CF_3_Ph	> 40	> 40	> 40	> 40	> 40
7	**4g**	H	H	N	N	3-CF_3_Ph	9.17 ± 2.02	20.12 ± 0.48	10.85 ± 2.22	9.86 ± 0.34	13.04 ± 1.27
8	**4h**	H	CH_3_	N	N	3-CF_3_Ph	9.31 ± 2.66	17.18 ± 3.40	12.44 ± 2.52	11.62 ± 2.08	11.06 ± 0.24
9	**4i**	H	CH_3_CH(OH)CH_2_	N	N	3-CF_3_Ph	13.08 ± 4.49	16.53 ± 3.53	9.15 ± 1.64	14.51 ± 2.19	11.62 ± 0.06
10	**4j**	4,5-(CH=CH–CH=CH)–	H	CH	N	3-CF_3_Ph	38.44 ± 3.95	24.79 ± 3.00	25.20 ± 3.35	18.09 ± 1.63	14.30 ± 0.66
11	**4k**	4,5-(CH=CH–CH=CH)–	CH_3_	CH	N	3-CF_3_Ph	> 40	35.30 ± 2.19	> 40	> 40	> 40
12	**4l**	3-CH_3_-5-Cl	H	C	CH	3-CF_3_Ph	> 40	> 40	> 40	> 40	> 40
13	**4m**	3-CH_3_-5-Cl	CH_3_	C	CH	3-CF_3_Ph	6.85 ± 0.23	6.77 ± 0.86	10.83 ± 1.02	21.44 ± 1.79	21.25 ± 3.59
14	**4n**	4,5-(CH=CH–CH=CH)–	H	CH	CH	3-CF_3_Ph	> 40	> 40	> 40	> 40	14.44 ± 0.01
15	**4o**	4,5-(CH=CH–CH=CH)–	CH_3_	CH	CH	3-CF_3_Ph	> 40	> 40	> 40	> 40	> 40
16	**5-FU** ^b^	**–**	**–**	**–**	**–**	**–**	8.02 ± 2.35	37.51 ± 3.25	24.75 ± 5.80	11.35 ± 1.67	8.65 ± 0.81

*NCI-H460* human large cell lung cancer cell line, *A549* human lung cancer cell line, *NCI-H1975* human lung cancer cell line, *HL-7702* human normal liver cells, *MDCK* Madin-Darby canine kidney cells

^a^IC_50_ compound concentration required to inhibit tumor cell proliferation by 50%

^b^5-Fluorouracil, used as a positive control

As shown in Table 1, the results further demonstrated that some of the synthesized nicotinamide-based diamides derivatives (**4d**, **4g**, **4h**, **4i**) exhibited higher inhibition activities compared to the control 5-FU under the same conditions. As indicated in Table 1, compound **4d** containing an alpha-aminoketone unit showed the strongest inhibitory effect on all three cell lines, with an IC_50_ values of 4.07 ± 1.30 (NCI-H460), 13.09 ± 2.45 (A549) and 12.82 ± 1.59 (NCI-H1975) μg/mL, respectively. However, the corresponding compound **4c** with an alpha-aminoalcohol moiety indicated lower inhibition activities. Meanwhile, the two groups of structurally similar compounds (**4a** and **4g**, **4b** and **4h**) exhibited significant activity differences, and the compounds **4g** and **4h** containing two nicotinamide units present good activities. Especially, we also can find that compounds **4g** presented obviously selective cytotoxic activities against the NCI-H460 and NCI-H1975 cell lines except A549 cell line. In addition, most of the similar diamides without nicotinamide moiety (entries 12–15) lost the inhibitory activity, however, compound **4m** exhibited good inhibition activities, which deserve the further studies. Furthermore, the cytotoxic effects on non-cancer cells HL-7702 and MDCK for these compounds have also been tested in our experiment. The results in Table 1 can further confirm that the cytotoxic effect of compound **4d** is more specific to cancer cells NCI-H460 compared to the control 5-FU, and the cytotoxic activities against NCI-H460 and normal cells (HL-7702 and MDCK) demonstrated that compound **4d** exhibited good selective cytotoxicity between NCI-H460 (IC_50_ = 4.07 ± 1.30 μg/mL) and normal cells (HL-7702, IC_50_ = 26.87 ± 0.95 μg/mL; MDCK, IC_50_ = 13.45 ± 0.29 μg/mL). These interesting finds may provide some useful information for developing potential cytotoxicity agents.

**Fig. 5:**
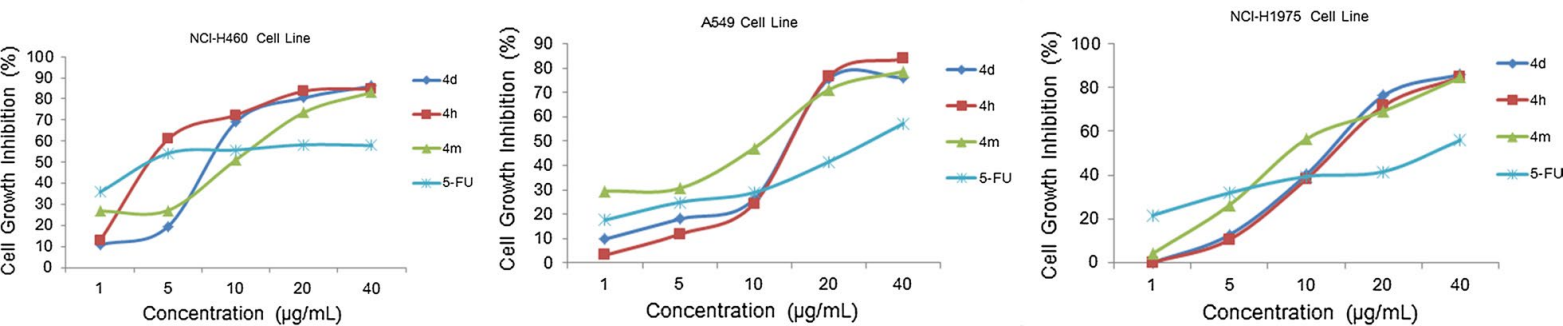
Dose-response analysis of cell growth inhibition activity for representative compounds **4d**, **4h**, **4m** and **5-FU** (positive control) against NCI-H460 (left), A549 (middle) and NCI-H1975 (right) cell lines

In addition, the dose-response analysis of cell growth inhibition activities for representation compounds **4d**, **4h**, **4m** and 5-FU have been displayed in Fig. 5, which identified that these compounds exhibited obvious cytotoxic effects on NCI-H460, A549, and NCI-H1975 cell lines in a concentration-dependent manner.

## Experimental

### Instrumentation and chemicals

^1^H-NMR spectra were recorded on a Bruker spectrometer at 600 (Bruker, Bremen, Germany) with DMSO-*d*_6_ as the solvent and TMS as the internal standard; ^13^C-NMR spectra were recorded on a Bruker spectrometer at 150 MHz with DMSO-*d*_6_ as the solvent. Chemical shifts are reported in *δ* (parts per million) values, and coupling constants ^n^ *J* are reported in Hz. Mass spectra were performed on a Waters ACQUITY UPLC® H-CLASS PDA (Waters®, Milford, MA, USA) instrument. Analytical thin-layer chromatography (TLC) was carried out on precoated plates, and spots were visualized with ultraviolet light. All chemicals or reagents used for syntheses were commercially available, were of AR grade, and were used as received.

### General synthetic procedures for the key intermediates **3**

The key intermediates **3** could be conveniently prepared by the coupling of substituted anthranilic acids with the N-substituted ortho-amino aryl acid according to the modified method. The general procedures are as follows: To a solution of substituted anthranilic acid **1** (1 mmol) and N-substituted ortho-amino aryl acid **2** (1 mmol) in 10 mL anhydrous acetonitrile was added pyridine (3 mmol), and then the reaction mixture was cooled to 0 °C. Where after, methanesulfonyl chloride (1.5 mmol) was added dropwise to the reaction mixture over 15–20 min. After addition, the reaction mixture was then allowed to warm to room temperature and stirred for additional hours, and which was detected by thin-layer chromatography. After completion of the reaction, the mixture was quenched by the addition of water and was stirred for 20 min. The suspended solid was collected by filtration and washed with water to afford the key intermediates **3** as pale yellow solids, which can be used directly for the next reaction without further purification.

### General synthetic procedures for target compounds **4a–k**

The typical process for these nicotinamide-based diamides derivatives is shown as following: To a solution of newly prepared intermediates **3** (1 mmol) in 8 mL of anhydrous acetonitrile was added the corresponding substituted amines (5 mmol) under nitrogen atmosphere. The clear solution was stirred at room temperature for several hours after which time TLC showed no remaining starting material. The reaction mixture was then concentrated in vacuum to remove the partial solvent, and then the residue was filtered and purified by silica gel column chromatography or recrystallization to give the target molecules. Their physico-chemical properties and the spectra data are as follows:

#### 2-(2-((3-(Trifluoromethyl)phenyl)amino)benzamido)nicotinamide **4a**

Yellowish powder, ^1^H NMR (600 MHz, DMSO-*d*_6_): *δ* = 11.72 (s, 1H), 9.29 (s, 1H), 8.48 (q, *J* = 7.2 Hz, 1H), 8.25 (bs, 1H), 8.11 (q, *J* = 6 Hz, 1H), 7.75 (d, *J* = 8 Hz, 1H), 7.71 (bs, 1H), 7.48–7.38 (m, 5H), 7.28 (q, *J* = 8.4 Hz, 1H), 7.21 (d, *J* = 8.4 Hz, 1H), 7.03 (t, *J* = 6 Hz, 1H); ^13^C NMR (150 MHz, DMSO-*d*_6_): *δ* = 169.59, 167.14, 150.64, 150.39, 143.60, 142.93, 137.89, 132.82, 130.84, 130.42, 129.79, 125.52, 123.72, 122.87, 122.13, 121.10, 120.72, 120.11, 117.44, 114.65; MS (ESI) *m/z* 423.3 (M + Na)^+^, calcd. for C_20_H_15_F_3_N_4_O_2_ m/z = 400.1.

#### N-Methyl-2-(2-((3-(trifluoromethyl)phenyl)amino)benzamido)nicotinamide **4b**

White powder, ^1^H NMR (600 MHz, DMSO-*d*_6_): *δ* = 12.25 (s, 1H), 9.18 (s, 1H), 8.75 (s, 1H), 8.47 (dd, *J* = 4.8 Hz, 1H), 8.03 (d, *J* = 7.8 Hz, 1H), 7.94 (d, *J* = 7.2 Hz, 1H), 7.74 (d, *J* = 7.8 Hz, 1H), 7.35–7.10 (m, 6H), 7.06–7.03 (m, 1H), 2.87 (d, *J* = 4.8 Hz, 3H); ^13^C NMR (150 MHz, DMSO-*d*_6_): *δ* = 167.80, 150.23, 149.96, 144.35, 143.73, 142.59, 137.60, 132.67, 132.49, 130.89, 130.82, 129.78, 123.38, 122.34, 121.81, 120.88, 120.26, 118.67, 117.66, 114.59, 26.68; MS (ESI) *m/z* 437.4 (M + Na)^+^, calcd. for C_21_H_17_F_3_N_4_O_2_ m/z = 414.1.

#### N-(2-Hydroxypropyl)-2-(2-((3-(trifluoromethyl)phenyl)amino)benzamido)nicotinamide **4c**

Yellowish powder, ^1^H NMR (600 MHz, DMSO-*d*_6_): *δ* = 11.40 (s, 1H), 9.58 (s, 1H), 8.66 (s, 1H), 8.48 (dd, *J* = 4.8 Hz, 1H), 8.07 (d, *J* = 7.8 Hz, 1H), 7.75 (d, *J* = 7.2 Hz, 1H), 7.50–7.02 (m, 9H), 3.80–3.75 (m, 1H), 3.20–3.08 (m, 2H), 1.02 (d, *J* = 6 Hz, 3H); ^13^C NMR (150 MHz, DMSO-*d*_6_): *δ* = 168.87, 152.22, 149.32, 143.61, 142.29, 141.58, 135.32, 132.75, 132.21, 130.84, 130.71, 129.34, 122.88, 122.03, 121.82, 120.71, 118.81, 118.12, 117.39, 115.06, 65.34, 47.60, 21.47; MS (ESI) *m/z* 481.3 (M + Na)^+^, calcd. for C_23_H_21_F_3_N_4_O_3_ m/z = 458.2.

#### N-(2-Oxopropyl)-2-(2-((3-(trifluoromethyl)phenyl)amino)benzamido)nicotinamide **4d**

Light brown powder, ^1^H NMR (600 MHz, DMSO-*d*_6_): *δ* = 11.18 (s, 1H), 9.78 (s, 1H), 8.74 (s, 1H), 8.58 (dd, *J* = 4.8 Hz, 1H), 8.12 (d, *J* = 7.8 Hz, 1H), 7.71 (d, *J* = 7.2 Hz, 1H), 7.58–7.12 (m, 8H), 4.12 (s, 2H), 2.06 (s, 3H); ^13^C NMR (150 MHz, DMSO-*d*_6_): *δ* = 169.63, 167.68, 156.76, 150.82, 145.13, 144.16, 142.42, 136.29, 134.15, 133.48, 131.32, 130.98, 129.64, 123.26, 122.86, 122.02, 120.96, 119.24, 118.78, 117.67, 114.25, 58.76, 25.35; MS (ESI) *m/z* 479.4 (M + Na)^+^, calcd. for C_23_H_19_F_3_N_4_O_3_ m/z = 456.1.

#### N-(2-Carbamoyl-4-chloro-6-methylphenyl)-2-((3-(trifluoromethyl)phenyl)amino)nicotinamide **4e**

Yellow powder, ^1^H NMR (600 MHz, DMSO-*d*_6_): *δ* = 10.45 (s, 1H), 10.30 (s, 1H), 8.41 (q, *J* = 7.2 Hz, 1H), 8.31 (s, 1H), 8.20 (q, *J* = 6 Hz, 1H), 7.97 (s, 1H), 7.88 (d, *J* = 6 Hz, 1H), 7.57–7.48 (m, 4H), 7.29 (d, *J* = 7.2 Hz, 1H), 7.01 (q, *J* = 7.2 Hz, 1H), 2.26 (s, 3H); ^13^C NMR (150 MHz, DMSO-*d*_6_): *δ* = 168.88, 166.89, 164.67, 154.03, 150.85, 147.17, 141.62, 139.07, 138.19, 133.14, 131.93, 131.20, 130.19, 126.15, 123.30, 118.94, 115.05, 114.37, 114.07, 106.68, 18.26; MS (ESI) *m/z* 471.2 (M + Na)^+^, calcd. for C_21_H_16_ClF_3_N_4_O_2_ m/z = 448.1.

#### N-(4-Chloro-2-methyl-6-(methylcarbamoyl)phenyl)-2-((3-(trifluoromethyl)phenyl)amino)nicotinamide **4f**

White powder, ^1^H NMR (600 MHz, DMSO-*d*_6_): *δ* = 10.45 (s, 1H), 10.16 (s, 1H), 8.68 (q, *J* = 7.2 Hz, 1H), 8.29 (s, 1H), 8.24 (q, *J* = 7.2 Hz, 1H), 7.92 (s, 1H), 7.59 (d, *J* = 6 Hz, 1H), 7.42–7.36 (m, 4H), 7.28 (d, *J* = 7.2 Hz, 1H), 7.01 (q, *J* = 7.2 Hz, 1H), 2.68 (d, *J* = 4.8 Hz, 3H), 2.27 (s, 3H); ^13^C NMR (150 MHz, DMSO-*d*_6_): *δ* = 171.22, 167.32, 166.90, 153.99, 150.66, 147.82, 141.67, 139.01, 138.11, 136.01, 133.38, 133.25, 131.77, 130.94, 130.78, 130.23, 128.95, 126.08, 124.79, 123.11, 117.26, 114.37, 24.71, 17.82; MS (ESI) *m/z* 485.2 (M + Na)^+^, calcd. for C_22_H_18_ClF_3_N_4_O_2_ m/z = 462.1.

#### N-(3-Carbamoylpyridin-2-yl)-2-((3-(trifluoromethyl)phenyl)amino)nicotinamide **4g**

Yellow powder, ^1^H NMR (600 MHz, DMSO-*d*_6_): *δ* = 11.26 (s, 1H), 9.24 (s, 1H), 8.57 (q, *J* = 7.2 Hz, 1H), 8.48 (s, 1H), 8.37 (q, *J* = 7.2 Hz, 1H), 7.96 (d, *J* = 7.2 Hz, 1H), 7.75–6.92 (m, 8H); ^13^C NMR (150 MHz, DMSO-*d*_6_): *δ* = 169.83, 168.78, 155.46, 153.23, 148.78, 144.02, 142.15, 138.33, 133.54, 131.62, 129.42, 127.55, 126.82, 123.67, 122.18, 121.37, 120.78, 118.46, 115.53; MS (ESI) *m/z* 424.5 (M + Na)^+^, calcd. for C_19_H_14_F_3_N_5_O_2_ m/z = 401.1.

#### N-Methyl-2-(2-((3-(trifluoromethyl)phenyl)amino)nicotinamido)nicotinamide **4h**

Yellowish powder, ^1^H NMR (600 MHz, DMSO-*d*_6_): *δ* = 11.22 (s, 1H), 9.12 (s, 1H), 8.61 (q, *J* = 7.2 Hz, 1H), 8.43 (s, 1H), 8.42 (q, *J* = 7.2 Hz, 1H), 7.84 (d, *J* = 7.2 Hz, 1H), 7.84–6.98 (m, 7H), 2.83 (d, *J* = 4.8 Hz, 3H); ^13^C NMR (150 MHz, DMSO-*d*_6_): *δ* = 169.54, 167.42, 156.65, 152.44, 149.56, 145.23, 143.23, 139.76, 133.86, 132.03, 128.62, 127.76, 127.02, 124.21, 122.76, 121.32, 120.22, 119.38, 114.17, 25.43; MS (ESI) *m/z* 438.4 (M + Na)^+^, calcd. for C_20_H_16_F_3_N_5_O_2_ m/z = 415.1.

#### N-(2-Hydroxypropyl)-2-(2-((3-(trifluoromethyl)phenyl)amino)nicotinamido)nicotinamide **4i**

Yellowish powder, ^1^H NMR (600 MHz, DMSO-*d*_6_): *δ* = 11.34 (s, 1H), 9.48 (s, 1H), 8.65 (q, *J* = 7.2 Hz, 1H), 8.43 (s, 1H), 8.32 (q, *J* = 7.2 Hz, 1H), 8.04 (d, *J* = 7.2 Hz, 1H), 7.89–6.98 (m, 8H), 3.86–3.77 (m, 1H), 3.28–3.14 (m, 2H), 1.08 (d, *J* = 6 Hz, 3H); ^13^C NMR (150 MHz, DMSO-*d*_6_): *δ* = 170.75, 156.35, 151.62, 144.72, 143.86, 141.92, 138.81, 133.42, 132.78, 131.65, 131.24, 129.86, 124.26, 122.77, 122.13, 118.94, 118.58, 116.37, 114.48, 63.38, 45.84, 21.22; MS (ESI) *m/z* 482.5 (M + Na)^+^, calcd. for C_22_H_20_F_3_N_5_O_3_ m/z = 459.2.

#### N-(3-Carbamoylnaphthalen-2-yl)-2-((3-(trifluoromethyl)phenyl)amino)nicotinamide **4j**

Yellowish powder, ^1^H NMR (600 MHz, DMSO-*d*_6_): *δ* = 12.81 (s, 1H), 10.82 (s, 1H), 8.99 (s, 1H), 8.66 (s, 1H), 8.54 (s, 1H), 8.47 (q, *J* = 6 Hz, 1H), 8.30 (s, 1H), 8.21 (q, *J* = 6 Hz, 1H), 8.00–7.92 (m, 4H), 7.66–7.63 (m, 1H), 7.56–7.54 (m, 2H), 7.33 (d, *J* = 7.2 Hz, 1H), 7.08 (q, *J* = 7.2 Hz, 1H); ^13^C NMR (150 MHz, DMSO-*d*_6_): *δ* = 171.64, 166.24, 154.66, 151.65, 141.33, 137.07, 135.55, 134.93, 130.36, 130.26, 129.20, 129.07, 127.74, 126.40, 125.84, 123.77, 123.58, 121.66, 118.20, 115.97, 115.39, 113.12; MS (ESI) *m/z* 473.2 (M + Na)^+^, calcd. for C_24_H_17_F_3_N_4_O_2_ m/z = 450.1.

#### N-(3-(Methylcarbamoyl)naphthalen-2-yl)-2-((3-(trifluoromethyl)phenyl)amino)nicotinamide **4k**

Yellowish powder, ^1^H NMR (600 MHz, DMSO-*d*_6_): *δ* = 12.50 (s, 1H), 10.76 (s, 1H), 9.11 (d, *J* = 3.2 Hz, 1H), 8.94 (s, 1H), 8.47 (q, *J* = 6 Hz, 1H), 8.43 (s, 1H), 8.29 (s, 1H), 8.21 (q, *J* = 6 Hz, 1H), 7.97–7.92 (m, 3H), 7.65–7.63 (m, 1H), 7.56–7.53 (m, 2H), 7.33 (d, *J* = 7.2 Hz, 1H), 7.11 (q, *J* = 7.2 Hz, 1H), 2.87 (d, *J* = 4.8 Hz, 3H); ^13^C NMR (150 MHz, DMSO-*d*_6_): *δ* = 169.53, 166.21, 154.56, 151.59, 141.37, 137.10, 135.02, 134.69, 130.25, 129.59, 129.20, 129.04, 128.96, 127.77, 123.71, 122.76, 118.55, 118.41, 115.94, 115.49, 113.29, 26.95; MS (ESI) *m/z* 487.2 (M + Na)^+^, calcd. for C_25_H_19_F_3_N_4_O_2_ m/z = 464.1.

### General synthetic procedures for compounds **4l–o**

In order to compare the activity, four similar diamides derivatives with no nicotinamide moiety have also been obtained according to the aforementioned method for target compounds. Their physico-chemical properties and the spectra data are as follows:

#### 5-Chloro-3-methyl-2-(2-((3-(trifluoromethyl)phenyl)amino)benzamido)benzamide **4l**

Yellowish powder, ^1^H NMR (600 MHz, DMSO-*d*_6_): *δ* = 10.12 (s, 1H), 9.28 (s, 1H), 7.94 (s, 1H), 7.80 (d, *J* = 7.2 Hz, 1H), 7.56 (s, 2H), 7.51–7.38 (m, 6H), 7.22 (d, *J* = 7.2 Hz, 1H), 7.02 (t, *J* = 7.2 Hz, 1H), 2.20 (s, 3H); ^13^C NMR (150 MHz, DMSO-*d*_6_): *δ* = 168.92, 167.57, 143.70, 142.61, 139.09, 135.40, 133.55, 132.53, 131.89, 130.88, 130.68, 129.98, 129.14, 126.03, 124.22, 122.71, 122.12, 120.64, 117.18, 114.49, 18.39; MS (ESI) *m/z* 470.2 (M + Na)^+^, calcd. for C_22_H_17_ClF_3_N_3_O_2_ m/z = 447.1.

#### 5-Chloro-N,3-dimethyl-2-(2-((3-(trifluoromethyl)phenyl)amino)benzamido)benzamide **4m**

White powder, ^1^H NMR (600 MHz, DMSO-*d*_6_): *δ* = 10.13 (s, 1H), 9.11 (s, 1H), 8.43 (d, *J* = 4.8 Hz, 1H), 7.74 (d, *J* = 7.8 Hz, 1H), 7.51–7.39 (m, 7H), 7.21 (d, *J* = 7.2 Hz, 1H), 7.03 (t, *J* = 7.2 Hz, 1H), 2.64 (d, *J* = 4.8 Hz, 3H), 2.22 (s, 3H); ^13^C NMR (150 MHz, DMSO-*d*_6_): *δ* = 167.57, 167.48, 143.80, 142.20, 139.02, 135.63, 133.42, 132.29, 131.79, 130.86, 130.73, 129.80, 125.88, 123.51, 121.76, 120.78, 117.33, 114.50, 26.58, 18.35; MS (ESI) *m/z* 484.3 (M + Na)^+^, calcd. for C_23_H_19_ClF_3_N_3_O_2_ m/z = 461.1.

#### 3-(2-((3-(Trifluoromethyl)phenyl)amino)benzamido)-2-naphthamide **4n**

Light brown powder, ^1^H NMR (600 MHz, DMSO-*d*_6_): *δ* = 12.32 (s, 1H), 9.45 (s, 1H), 9.06 (d, *J* = 4.8 Hz, 1H), 8.92 (s, 1H), 8.47 (s, 1H), 7.96 (q, *J* = 7.2 Hz, 2H), 7.84 (dd, *J* = 8.4 Hz, 1H), 7.67–7.54 (m, 1H), 7.48–7.36 (m, 7H), 7.21 (d, *J* = 7.8 Hz, 1H), 7.11–7.05 (m, 1H); ^13^C NMR (150 MHz, DMSO-*d*_6_): *δ* = 169.12, 165.55, 144.15, 142.92, 136.82, 134.47, 133.18, 131.25, 130.48, 129.85, 129.24, 128.74, 128.32, 127.75, 125.47, 124.18, 122.92, 121.74, 121.38, 121.07, 118.82, 118.05, 114.51, 99.62; MS (ESI) *m/z* 472.3 (M + Na)^+^, calcd. for C_25_H_18_F_3_N_3_O_2_ m/z = 449.1.

#### N-Methyl-3-(2-((3-(trifluoromethyl)phenyl)amino)benzamido)-2-naphthamide **4o**

White powder, ^1^H NMR (600 MHz, DMSO-*d*_6_): *δ* = 12.15 (s, 1H), 9.35 (s, 1H), 9.01 (d, *J* = 4.8 Hz, 1H), 8.96 (s, 1H), 8.35 (s, 1H), 7.90 (q, *J* = 7.8 Hz, 2H), 7.80 (dd, *J* = 9.6 Hz, 1H), 7.62–7.59 (m, 1H), 7.52–7.40 (m, 6H), 7.19 (d, *J* = 7.2 Hz, 1H), 7.14–7.11 (m, 1H), 2.80 (d, *J* = 4.2 Hz, 3H); ^13^C NMR (150 MHz, DMSO-*d*_6_): *δ* = 169.34, 166.86, 143.91, 142.71, 135.31, 134.69, 132.99, 130.79, 129.37, 129.17, 128.91, 128.84, 127.66, 126.18, 123.52, 122.66, 121.93, 121.51, 121.29, 118.75, 117.71, 114.62, 99.95, 26.79; MS (ESI) *m/z* 486.3 (M + Na)^+^, calcd. for C_26_H_20_F_3_N_3_O_2_ m/z = 463.2.

### In vitro cytotoxicity assays

The in vitro cytotoxicity of the synthesized compounds against different human lung cancer cell lines (NCI-H460, A549, NCI-H1975) and normal cells (HL-7702 and MDCK) were measured with the 3-(4,5-dimethylthiazol-2-yl)-2,5-diphenyl tetrazolium bromide (MTT) assay. All the data of the experiment were analyzed with SPSS software, and the 50% inhibitory concentrations (IC_50_) of each compound for the different cell lines were determined. A control was run for each test, and all assays were performed in triplicate on three independent experiments, and measurement data were expressed as the mean ± SD.

## Conclusion

In summary, a series of diamides derivatives based on nicotinamide scaffold have been conveniently prepared and evaluated as potential cytotoxic agents. The bioassay indicated that some of these newly synthesized compounds exhibited good cytotoxic activities. Especially, the most potent compounds **4d** and **4h** exhibited higher cytotoxic activities against the tested lung cancer cell lines as compared with 5-FU in vitro, and these interesting results might be used to develop novel lead molecules for potential anticancer agents.
